# Development of a LINAC head model for the CyberKnife VSI‐System using EGSnrc Monte Carlo system

**DOI:** 10.1002/acm2.14137

**Published:** 2023-09-15

**Authors:** Martin Thiele, Kirsten Galonske, Iris Ernst, Andreas Mack

**Affiliations:** ^1^ German CyberKnife‐Center Soest Germany; ^2^ German Center for Stereotaxy and Precision Irradiation Soest Germany; ^3^ Swiss Neuro Radiosurgery Center Zurich Switzerland

**Keywords:** CyberKnife, EGSnrc, Monte Carlo simulation

## Abstract

**Introduction:**

In order to understand the interaction processes of photons and electrons of the CyberKnife VSI‐System, a modeling of the LINAC head must take place. Here, a Monte Carlo simulation can help. By comparing the measured data with the simulation data, the agreement can be checked.

**Materials and methods:**

For the Monte Carlo simulations, the toolkit EGSnrc with the user codes BEAMnrc and DOSXZYnrc was used. The CyberKnife VSI‐System has two collimation systems to define the field size of the beam. On the one hand, it has 12 circular collimators and, on the other, an IRIS‐aperture. The average energy, final source width, dose profiles, and output factors in a voxel‐based water phantom were determined and compared to the measured data.

**Results:**

The average kinetic energy of the electron beam for the CyberKnife VSI LINAC head is 6.9 MeV, with a final source width of 0.25 cm in x‐direction and 0.23 cm in y‐direction. All simulated dose profiles for both collimation systems were able to achieve a global gamma criterion of 1%/1 mm to the measured data. For the output factors, the deviation from simulated to measured data is < 1% from a field size of 12.5 mm for the circular collimators and from a field size of 10 mm for the IRIS‐aperture.

**Conclusion:**

The beam characteristics of the CyberKnife VSI LINAC head could be exactly simulated with Monte Carlo simulation. Thus, in the future, this model can be used as a basis for electronic patient‐specific QA or to determine scattering processes of the LINAC head.

## 1 INTRODUCTION

Monte Carlo methods are stochastic procedures, which calculate with random numbers, and was founded by the pioneers Stanislaw Ulam and Nicholas Metropolis in the 1940s.^1^ For example, electrons or photons and their interactions in matter can be simulated. The first paper in medical physics using electron transport by Monte Carlo method comes from Robert R. Wilson.^2^ Now‐a‐days, the Monte Carlo method is the gold standard in radiation therapy, since it provides, among other things, an accurate dose calculation in inhomogeneous materials.^3^

**FIGURE 1:**
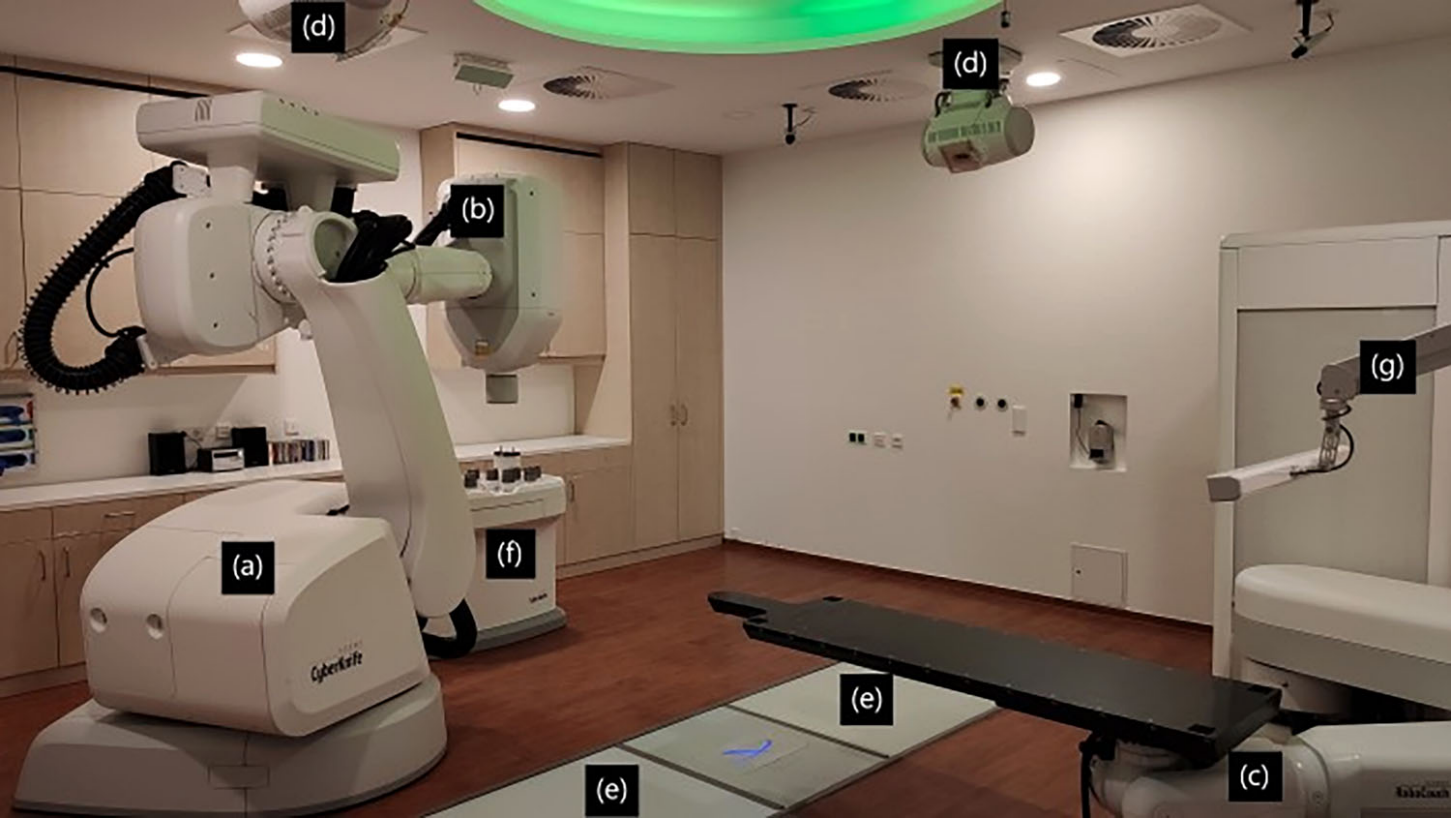
The CyberKnife VSI‐System in Soest, Germany: (a) industrial robot, (b) linear accelerator, (c) treatment table, (d) x‐ray tubes, (e) flat panel detectors, (f) collimator table, and (g) camera system.

The CyberKnife VSI‐System (Accuray, Sunnyvale, CA, USA) (Figure 1), where VSI stands for versatile, simple, and intelligent, is an advanced radiotherapy device for stereotactic radiosurgery (SRS) and stereotactic body radiotherapy (SBRT)^4^ and was developed by John R. Adler.^5^ It consists of the following components^6^:

- the linear accelerator (LINAC), which is flanged to an industrial robot manufactured by KUKA (Augsburg, Germany),
- the treatment table, also called RoboCouch,
- two x‐ray tubes (VAREX Imaging, Salt Lake City, Utah, USA) with two Flat Panel Detectors (Perkin Elmer, Waltham, Massachusetts, USA) facing each other for orthogonal verification images for patient alignment,
- a collimator table, also called XChange table, with 12 different circular collimators and an IRIS‐aperture,^7^ and
- a camera system for breath‐triggered irradiation

The LINAC is a 9.5 GHz X‐band accelerator with 6 MV without flattening filter. The dose rate is 800 MU/min (where MU is equal to monitor unit). The six‐axis industrial robot can perform non‐coplanar irradiations with up to 1200 irradiation positions per field size. For collimation of the beam field, 12 circular collimators (diameters: 5 , 7.5 , 10 , 12.5 , 15 , 20 , 25 , 30 , 35 , 40 , 50, and 60 mm defined 800 mm SAD) and an IRIS‐aperture, which can approach the same diameters as the circular collimators, are available.

Research papers have already been published on the creation of a Monte Carlo model for the CyberKnife LINAC head.^8^, ^9^, ^10^, ^11^ These report on the circular collimators as well as the InCise multileaf collimator (MLC).^12^ However, the IRIS‐aperture is missing. In the present work, using the Electron Gamma Shower (EGS) software package,^13^ further developed at the National Research Council (NRC) in Canada, a more accurate Monte Carlo model is created for the CyberKnife VSI‐System, which will be used for plan verification in the future.

## 2 MATERIAL AND METHODS

In order to be able to check the modeling of the CyberKnife VSI LINAC head, beam data [percentage depth dose curves (PDD), dose profiles (DP), and output factors (OF)] are required. These were measured using a water phantom (type MP3, Fa. PTW, Freiburg, Germany). The size of the water phantom is 29 cm x 29 cm x 40 cm. The two‐channel electrometer PTW Tandem (type 10011), the TBA Control Unit and a hand control were connected to the water phantom as a measuring system. In the irradiation field the diode E type PTW 60017 (unshielded diode) was placed in the water phantom and the semiflex chamber type PTW 31010 as reference chamber directly at the CyberKnife LINAC head. The MP3 water phantom and the measuring system were controlled outside the irradiation room using the MEPHYSTO mc^2^ software, version 3.3.17 (PTW, Freiburg, Germany).

Regarding to the reference conditions, a SSD of 800 mm applies to PDDs, and a SAD of 800 mm applies to DPs and OFs in a water depth of 15 mm. The measured OFs were multiplied by the correction factor $k_{Q_{clin},\;Q_{msr}}^{f_{clin},\;f_{msr}}$ from TG 483.^14^

The size and what material each component of the CyberKnife VSI LINAC head is made of is from Accuray (Accuray Incorporated). The original size for secondary collimators was adjusted where appropriate if the deviation in half‐width (FWHM) from measured data to simulated data was > 1%. The 5 mm field size was not adjusted because it is not used for irradiation on patients at the German CyberKnife‐Center in Soest.

In the 1970s, the Electron Gamma Shower (EGS) software package was developed at the Stanford Linear Accelerator Center (SLAC). With improvements and further development of the program code by the National Research Council (NRC) in Canada, this is now called EGSnrc. With EGSnrc, Monte Carlo simulations are possible in which photons, electrons, and positrons penetrate matter with kinetic energy from 1 keV to 10 GeV.^15^ The interaction processes that can be simulated with this software toolkit can be found in “The EGSnrc Code System.^13^” EGSnrc provides the user code BEAMnrc^16^ for modeling the CyberKnife VSI LINAC head, producing a phase space file at the end of the simulation that is subsequently used for dose depositions in the water phantom in the user code DOSXYZnrc.^17^ The parameters of the electron beam were adjusted until the measured data matched the simulation data.

**FIGURE 2:**
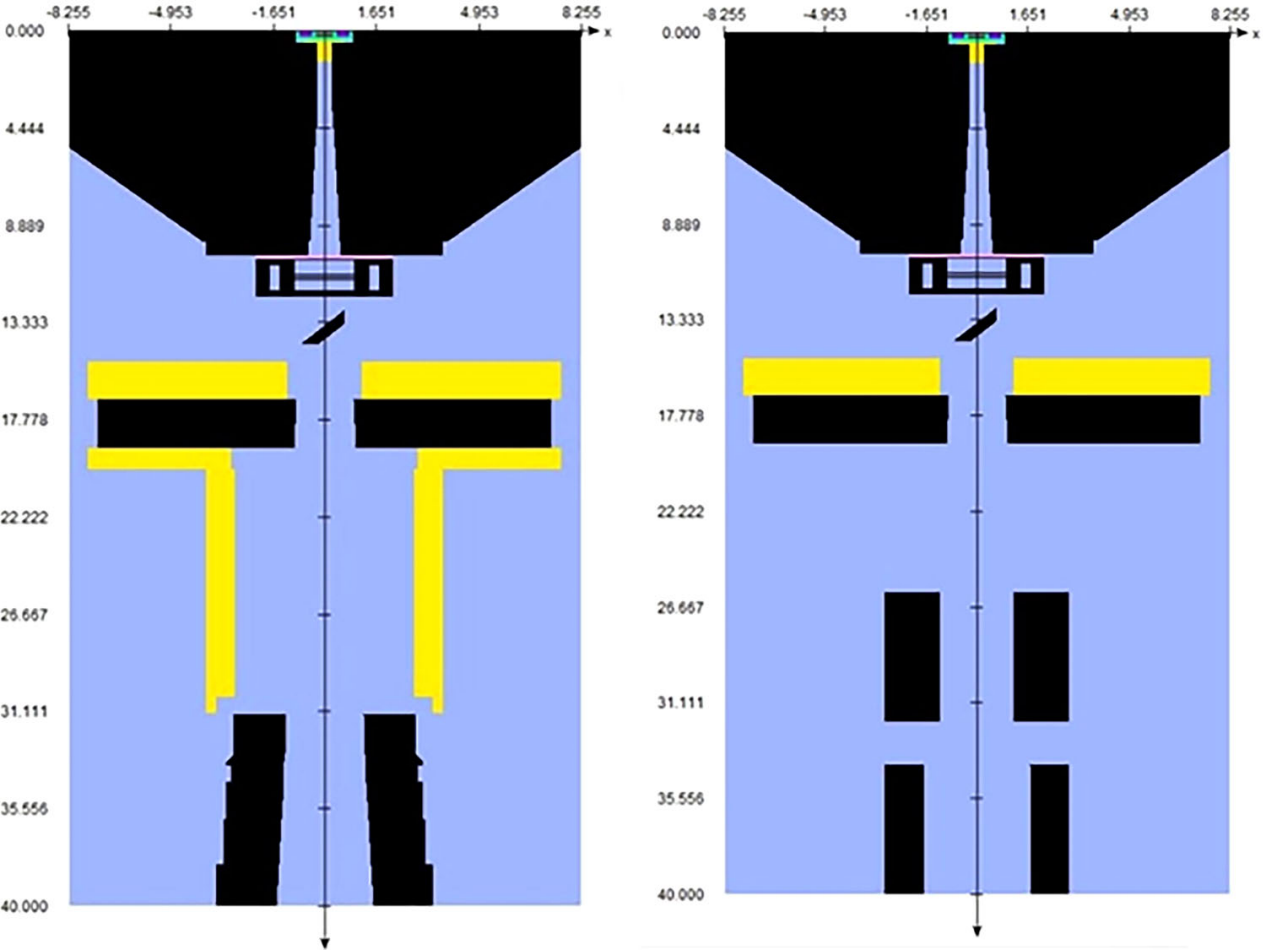
All composited component modules make up the complete CyberKnife VSI LINAC head for the circular collimators on the left and the IRIS‐aperture on the right.

In BEAMnrc, the CyberKnife VSI LINAC head is simulated. By stringing together, the individual component modules (CM), which have the size and material composition of each individual component, the finished LINAC head is created (Figure 2). Table 1 lists the component modules for the CyberKnife VSI LINAC head with circular collimator and IRIS‐aperture.

**TABLE 1:** Overview of the component modules for the CyberKnife VSI LINAC head with circular collimator and IRIS‐aperture.

Circular collimator		IRIS‐Aperture	
CM	Identifier	CM	Identifier
FLATFILT	Target	FLATFILT	Target
CONS3R	PriColI	CONS3R	PriColI
FLATFILT	PriColII	FLATFILT	PriColII
SLABS	PbFilter	SLABS	PbFilter
CHAMBER	IonChamb	CHAMBER	IonChamb
MIRROR	AlMirror	MIRROR	AlMirror
FLATFILT	MstTool	FLATFILT	MstTool
FLATFILT	PtSld	FLATFILT	PtSld
FLATFILT	PtPrt	BLOCK	BankUp
FLATFILT	SecCol	BLOCK	BankLow

**FIGURE 3:**
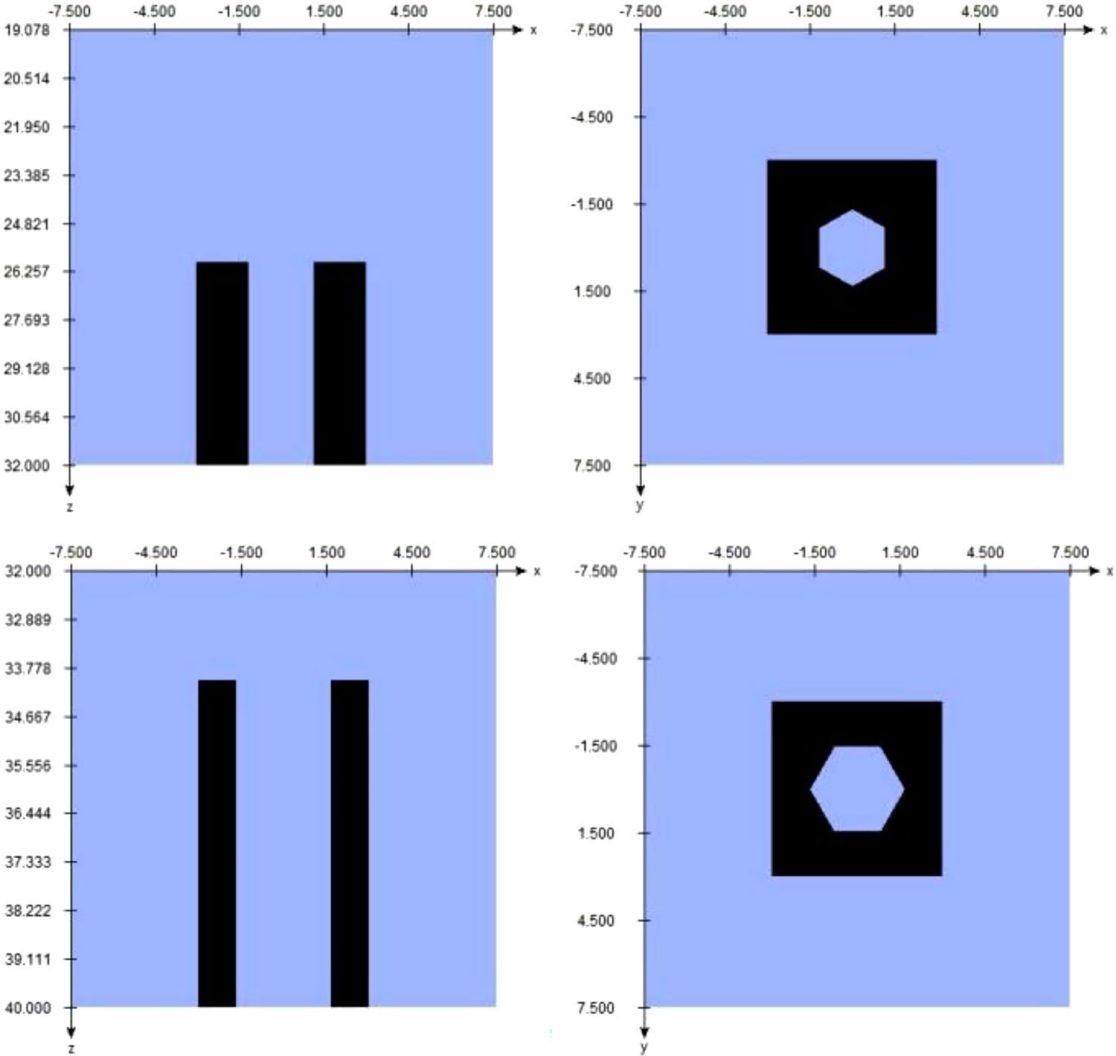
Component modules for the 60 mm IRIS‐aperture. Top: Component module for the upper bank (BankUp), Bottom: Component module for the lower bank (BankLow).

The IRIS‐aperture can replicate the field sizes of the circular collimators. It is divided into an upper and a lower bank, each with six segments made of a tungsten‐copper alloy. The banks are rotated 30° to each other, which results in a dodecagonal field aperture.^7^ In BEAMnrc the IRIS‐aperture can be modeled with two BLOCK component modules (Figure 3).

### 2.1 Adjustment of the full width at half maximum

After a Monte Carlo simulation of the dose profiles, the half‐width was evaluated for each collimator. In case of a deviation > 1% between the measured data and the simulated data, a geometrical adjustment of the component module secondary collimator for circular collimators or of the component modules lower and upper bank for the IRIS™‐aperture was performed. For this purpose, the intercept theorem was applied. The distance from the secondary collimator end to the measurement point is known and is 40 cm. Through Monte Carlo simulation, the field width (FW) is obtained. This is halved to obtain the field size radius. Using the equation below, the distance *z* from the source to the end of the secondary collimator can be calculated:

(1) $$z\;=\;\frac{FW_{Radius\;from\;manufacturer}\;\times \;\left(40\;cm\;+\;z\right)}{FW_{Radius\;from\;MC}}$$

By summing *z* and 40 cm, the total length (SAD) is obtained. Thus, the new radius *r* for the secondary collimator for the new Monte Carlo simulation is as follows:

(2) $$FW_{Radius\;for\;MC_{new}}=\frac{FW_{Radius\;from\;measuremenet}\times \;z}{\left(40\;cm\;+\;z\right)}$$

Equations 1 and 2 help to adjust the field sizes for the circular collimators. For field sizes > 7.5 mm, a new radius must be calculated for each of the seven layers in the component module. For the IRIS‐aperture, the lower opening of each bank is specified by the manufacturer. The radius for the 60 mm field size is 1.5 cm for the lower bank at a distance of 40 cm from the radiation source and 1.2 cm for the upper bank at a distance of 32 cm from the radiation source. The radius is then divided by the cosine of 30° to obtain the diagonal from the central axis to the corner point for the component module. Afterwards the length between the corner point and the radius can be calculated with the help of the Pythagorean theorem. The values of the corner points for the other bank must be rotated by 30° to correspond to the IRIS‐aperture. The following rotation matrix helps for this:

(3) $$R_{\alpha }=\left(\begin{array}{cc} \cos\alpha & -\sin\alpha \\ \sin\alpha & \cos\alpha \end{array}\right)$$

To check the calculated field size, the values of the corner points of the upper bank, for example, can be multiplied by the factor 400/320. All corner points of the upper and lower bank thus lie in one plane. By connecting the individual corner points in a spreadsheet program, the field size can be seen. The field size can be determined by Monte Carlo simulation. The desired radius results from the above equations.

### 2.2 Definition of the radiation source in BEAMnrc

To define a radiation source in BEAMnrc there are several radiation source routines.^16^ The beam source routine ISOURC = 19 is closest to the CyberKnife VSI‐System. Thus, an elliptical beam with a Gaussian distribution in x and y directions, parallel or with angular spread is possible. At the beginning of the Monte Carlo simulation, the kinetic energy of the beam was set to monoenergetic 6.6 MeV with a FWHM of 0.24 cm in x‐direction and 0.20 cm in y‐direction and an angular spread of 0 °.

### 2.3 Transport parameters in BEAMnrc

The calculation of the particle transport was performed with the recommended standard parameters in order to keep the calculation time short while maintaining accuracy. As global lower threshold energy for the electron transport (ECUT) 0.7 MeV and for the photon transport (PCUT) 0.01 MeV is used. If the energy falls below this threshold, no further interactions will take place and the energy will be deposited at the interaction site. The PRESTA‐I algorithm is used to calculate how electrons behave at material transitions. PRESTA stands for Parameter Reduced Electron Step Transport Algorithm. For the calculation of lateral and longitudinal corrections to account for elastic scattering in a so‐called condensed history step,^18^ the PRESTA‐II algorithm is used. Condensed History Technique (CHT) now‐a‐days divides the interaction processes of charged particle fates into hard and soft collisions as well as hard and soft bremsstrahlung production.^2^ The maximum global step size is set to 5 cm. The electron spin effect is turned on to get a good calculation for the backscattering. However, electron collision ionization is not used. If bremsstrahlung photons or pair productions are generated, the bremsstrahlung angle is calculated according to the Koch‐Motz distribution. This is done via the Simple setting. More details about this calculation can be found in the report PIRS‐0203 Improved bremsstrahlung photon angular sampling in the EGS4 code system by Bielajew, A. F., Mohan R., and Chui C. S. of the National Research Council of Canada. In the pair production, only the first term of the Motz equation is used. The effective cross section for the bremsstrahlung as well as for the pair production is calculated according to the Bethe‐Heitler (BH) formalism. To calculate differential cross sections for Compton scattering, the Klein‐Nishina formula is used. Furthermore, it was determined that the photoelectron takes the direction of the incident photon. The Rayleigh scattering is significant in the very low keV range (about 20 keV) and can thus be neglected for the CyberKnife, which operates in the MeV range (6 MeV). The atomic relaxation in its ground state after the photo‐, Compton effect as well as ionization process is eliminated. The effective cross sections of the materials used for the Monte Carlo simulation for photons were retrieved from the XCOM: Photon Cross Sections database at the National Institute of Standards and Technology (NIST) and saved as a *.pegs4dat file. There is no output of the photon cross sections. A short overview, which transport parameters are used for the Monte Carlo simulations shown here, is given in Table 2.

**TABLE 2:** Defined transport parameters for electrons and photons in BEAMnrc for the Monte Carlo simulations.

Electrons		Photons	
ECUT	0.7 MeV	PCUT	0.01 MeV
Boundary crossing algorithm	PRESTA‐I	Bound Compton scattering	Off
Electron‐step algorithm	PRESTA‐II	Compton cross‐sections	Default
Spin effects	On	Pair angular sampling	Simple
Electron impact ionization	Off	Pair cross‐sections	BH
Brems angular sampling	Simple	Photoelectron angular sampling	Off
Brems cross‐sections	BH	Rayleigh scattering	Off
		Atomic relaxations	Off
		Photon cross‐sections	XCOM
		Photon cross‐sections output	Off

### 2.4 Variance reduction techniques

Variance reduction techniques are used in the Monte Carlo simulations to keep them as effective as possible. In BEAMnrc, directed bremsstrahlung splitting (DBS), the Russian roulette technique and photon forcing are used. The Monte Carlo simulations in DOSXYZnrc are performed with DBS, Russian roulette technique, and range rejection.

### 2.5 Number of histories

For the Monte Carlo simulations in BEAMnrc 1 × 10^8 and in DOSXYZnrc 1 × 10^9 number of histories were used. The energy deposition in each voxel is subject to a statistical uncertainty s, which usually decreases with $N^{-\frac{1}{2}}$,^13^ where *N* is the number of histories simulated.

### 2.6 Determination of the mean kinetic energy of the electron beam

The mean kinetic energy for the modeled CyberKnife VSI LINAC head is needed to perform the Monte Carlo simulation with the exact energy of the central beam. To determine the average kinetic energy, several depth dose curves with different monoenergetic energies were simulated in EGSnrc (see^19^). The increment was increased by 0.1 MeV, starting at 6.6 MeV to 7.0 MeV. The central beam hits perpendicularly on the self‐generated voxel‐based water phantom, which was created in DOSXYZnrc. The dimensions of the water phantom are 29 cm x 29 cm x 32 cm. The edge area of the water phantom in the x‐ and y‐direction is 8 cm, and the area of the central beam is 13 cm. For high resolution in the central beam area, the 13 cm was divided into 0.2 cm. In z‐direction, starting at 0 cm, the first 2 cm are divided into 0.1 cm, the further 8 cm into 0.2 cm and the last 22 cm into 0.5 cm. Thus, a high resolution is given especially in the build‐up area of the photon beam. The SSD is 800 mm during the Monte Carlo simulation. The 60 mm circular collimator was used as the secondary collimator. The parameters for the simulation of the depth dose curves include the average energy and the focal spot size. However, there is hardly any parameter which would have an influence on the depth dose curve.^20^ This is because the particles tend to deposit their energy near the central beam. The average kinetic energy also has an influence on the dose profile. Therefore, the final average kinetic energy results from the relationship between the depth dose curve and the dose profile. The Monte Carlo simulations were divided into six to eight jobs and computed on a computer with 3.7 GHz Intel(R) Core (TM) i7‐4820K processor, 16 GB memory with four cores each (eight logical processors). The computation time in EGSnrc depends on the number of histories and is about two days for the Monte Carlo simulation for the depth dose curves.

To analyze the depth dose curves, first the phantom file (*.egsphant) and the 3D dose distribution file (*.3ddose) were imported into the dose viewer VICTORIA (Voxel Interactive Contour Tool for Online Radiation Intensity Analytics).^21^ In the central beam, coordinate origin [x = 0, y = 0], the depth dose curve could be exported as a *.csv file. Using the gamma analysis software ScanDoseMatch (version 1.5.15, Fa. QXRay Consulting, Forest Hill, Maryland, USA), the *.csv of the Monte Carlo simulation was compared with the *.csv file of the measurement to determine the local gamma criterion. The local gamma criteria 3%/3 mm, 2%/2 mm, and 1%/1 mm with a spline SF of 10 were investigated at a measurement depth of 0.1 to 30 cm, and the passing rate (PR) was analyzed.

### 2.7 Determination of the final source width

The final source width can be verified by a Monte Carlo simulation of a dose profile. The 60 mm circular collimator was chosen as reference for the source width. In EGSnrc, the source width can be defined for radiation sources routine ISOURC = 19 via the half‐width (FWHM). Here a Gaussian distribution in x‐ and y‐direction is given. Initially, the half‐width was set to 0.2 cm in the x‐direction and 0.0 cm in the y‐direction. An increment of 0.01 cm in the x‐direction was chosen up to an x‐value of 0.27 cm. Meanwhile, the value for the y‐direction remained at 0.0 cm. The central ray impinges perpendicularly on the self‐generated voxel‐based water phantom. The dimensions of the water phantom are 31.75 cm x 31.75 cm x 32 cm. In x‐, y‐, and z‐direction, the voxels are 0.25 cm in size. This corresponds to a volume of ≈ 0.016 cm^3^ per voxel. Thus, the dose can be determined in high resolution even in the edge region of the dose profile. The SAD is 800 mm, and the SSD is 785 mm. A dose profile is influenced by parameters, such as the average energy of the incident electron beam with a Gaussian distribution. Further influences in a Monte Carlo simulation of dose profiles can be found in the AAPM (American Association of Physicists in Medicine) Report 105.^20^ The Monte Carlo simulations of dose profiles were performed as described for the determination of the mean kinetic energy of the electron beam.

The simulated dose profiles were imported into VICTORIA. In the central beam, coordinates [x = 0 cm, y = 0 cm, z = 1.5 cm], the dose profile for the x‐ and y‐axes could now be exported. Using ScanDoseMatch the local gamma criteria 3%/3 mm, 2%/2 mm, and 1%/1 mm with a spline SF of 10 at an evaluation range of −3.2  to 3.2 cm (This corresponds to the penumbra width 80%−20% of the circular collimator 60 mm—at a water depth of 15 mm and a SAD of 80 cm) were examined and the passing rate was analyzed. After determining the final source width in the x‐direction, a FWHM of 0.21 cm in the y‐direction was started and increased by 0.01  to 0.27 cm. Finally, a gamma analysis is performed with the same gamma criteria as in the x‐direction; additionally, the gamma criterion 0.5%/0.5 mm.

### 2.8 Monte Carlo simulation of dose profiles for circular collimators and IRIS‐aperture

After the average kinetic energy of the central beam (6.9 MeV) and the final source width (0.25 cm in x‐direction and 0.23 cm in y‐direction) have been determined, the Monte Carlo simulations for the respective collimator sizes can now be performed. The Monte Carlo simulations were performed in a self‐made voxel‐based water phantom with the dimensions 31.75 cm x 31.75 cm x 32 cm. In the x, y, and z directions, the voxels are 0.25 cm in size. This corresponds to a volume of ≈ 0.016 cm^3^ per voxel. Thus, the dose can be determined in high resolution even in the edge region of the dose profile. The measurement depth is 15 mm with an SSD of 785 mm. The central beam hits the water phantom vertically. As described earlier in this paper, there are some parameters that can influence the dose profiles (see^20^). The Monte Carlo simulations were carried out as shown above.

The simulated dose profiles were imported into VICTORIA. In the central beam, coordinates [x = 0 cm, y = 0 cm, z = 1.5 cm], the dose profile for the x‐ and y‐axes could now be exported. ScanDoseMatch was used to examine the global gamma criteria 3%/3 mm, 2%/2 mm, and 1%/1 mm with a spline SF of 10 over the entire dose profile and to analyze the passing rate. In the course of this work, it was found that the gamma criterion of 3%/3 mm was always achieved. For this reason, the gamma criterion 3 %/3 mm was not investigated for the IRIS‐aperture. In addition, the determination of the half‐width (FWHM) was also performed. By comparing the measured dose profiles with the simulated dose profiles, a deviation should not be greater than 1%. If it did, a field size adjustment took place and it had to be simulated again in EGSnrc.

### 2.9 Monte Carlo simulation of the output factors

To complete the modeling of the CyberKnife VSI LINAC head using EGSnrc, output factors of the two collimation systems must be simulated. These were determined with a constant SAD of 800 mm and an SSD of 785 mm. A self‐created voxel‐based water phantom is used here with dimensions of 7.1 cm x 7.1 cm x 3.2 cm. The voxel of interest in 15 mm water depth in the central beam has a size of 0.0005 cm^3^ (x‐direction 0.1 cm, y‐direction 0.1 cm, and z‐direction 0.5 cm). The central beam hits the water phantom perpendicularly. The Monte Carlo simulations were carried out, as mentioned above.

The simulated output factors were imported into VICTORIA. In the central beam, coordinates [x = 0 cm, y = 0 cm, z = 1.5 cm], the output factor could now be determined. Subsequently, the output factor had to be normalized to the 60 mm collimator. For the IRIS‐aperture, the output factors must also be normalized to the 60 mm collimator and then multiplied by the IRIS to circular collimator ratio. The IRIS to circular collimator ratio is obtained from a water phantom measurement with both collimation systems with a field size of 60 mm. The SAD is 800 mm, and the water depth is 15 mm. This procedure was chosen in order to correspond to the measured beam data.

### 2.10 Uncertainties in the Monte Carlo simulations

Variance reduction techniques are used in Monte Carlo simulations to save computation time. The variance is directly related to the number of histories and thus describes a certain statistical uncertainty. This type of uncertainty belongs to type A of standard uncertainties. The higher the number of histories is chosen, the closer the statistical uncertainty converges to 0. Since the variance of the number of histories is difficult to calculate because the true value is unknown, the estimated variance *s(N)* during a Monte Carlo simulation can be calculated as follows^2^:

(4) $$s\left(N\right)\;=\;\sqrt{\frac{\langle f{}^{2}\left(N\right)\rangle \;-\;{\langle f\left(N\right)\rangle }^{2}}{N\;-\;1}}$$

where *N* is the number of histories, ⟨f(N)⟩ is the calculated mean around the true value *f* , and $\left\langle f^{2}\left(N\right)\right\rangle$ is the calculated mean of *f* ^2^ of the Monte Carlo simulation. Using the history by history method, the type A uncertainty during a Monte Carlo simulation can be estimated in BEAMnrc and DOSXYZnrc^20^, ^22^ Care was taken to ensure that the statistical uncertainty in the Monte Carlo simulation was ≤ 0.5% for the depth dose curves, dose profiles, and output factors. Type B standard uncertainties can occur due to the modeling of the CyberKnife VSI LINAC head. That is, components do not have the correct material properties or the geometric relationship between the individual components is inaccurate. Furthermore, inaccuracies may occur due to the selected transport parameters.

### 2.11 Gamma analysis

The gamma analysis, also called gamma index method, allows the comparison of two dose distributions in 2D or 3D space. For the gamma analysis the software ScanDoseMatch is used. This uses the same formulas as a basis for calculation as Low et al. has already explained.^23^ The Gamma Index considers two criteria, on the one hand the difference of the dose and on the other hand the deviation of the distance (Distance‐to‐Agreement—DTA) from a measured to a calculated point. The result of the Gamma Index method, how many examined points have met the acceptance criteria, is expressed by the passing rate in percent.^24^

## 3 RESULTS

### 3.1 Energy determination of the CyberKnifes VSI LINAC head

**FIGURE 4:**
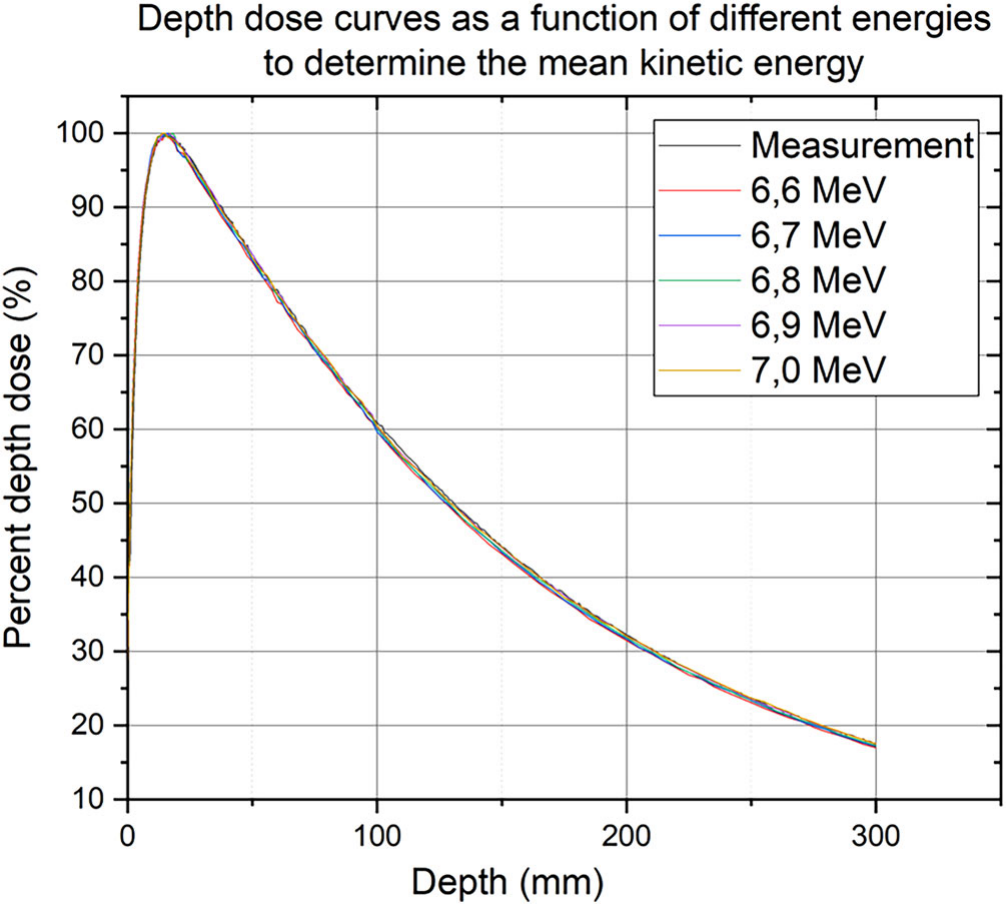
Comparison of the depth dose curve of the measurement with the simulated depth dose curves from 6.6 to 7.0 MeV for the energy determination of the CyberKnife VSI LINAC head.

Through several Monte Carlo simulations of depth dose curves with the 60 mm circular collimator and a constant SSD of 800 mm with different energies, the average kinetic energy of the CyberKnife VSI LINAC head can be determined for the time being. Figure 4 shows the simulated depth dose curves versus the measurement. All simulated depth dose curves are close to the measured curve. The statistical uncertainty in the central beam is ≤ 0.4% for all depth dose curve simulations.

Table 3 lists the results of the gamma analysis of the comparison of the simulated depth dose curves with different energy. Only 6.9 MeV met the local gamma criterion of 1%/1 mm.

**TABLE 3:** Results of gamma analysis of the comparison of depth dose curves as a function of energy.

E (MeV)	3%/3 mm	2%/2 mm	1%/1 mm
6.6	100	76	39
6.7	100	99	55
6.8	100	100	73
6.9	100	100	100
7.0	100	100	97

**FIGURE 5:**
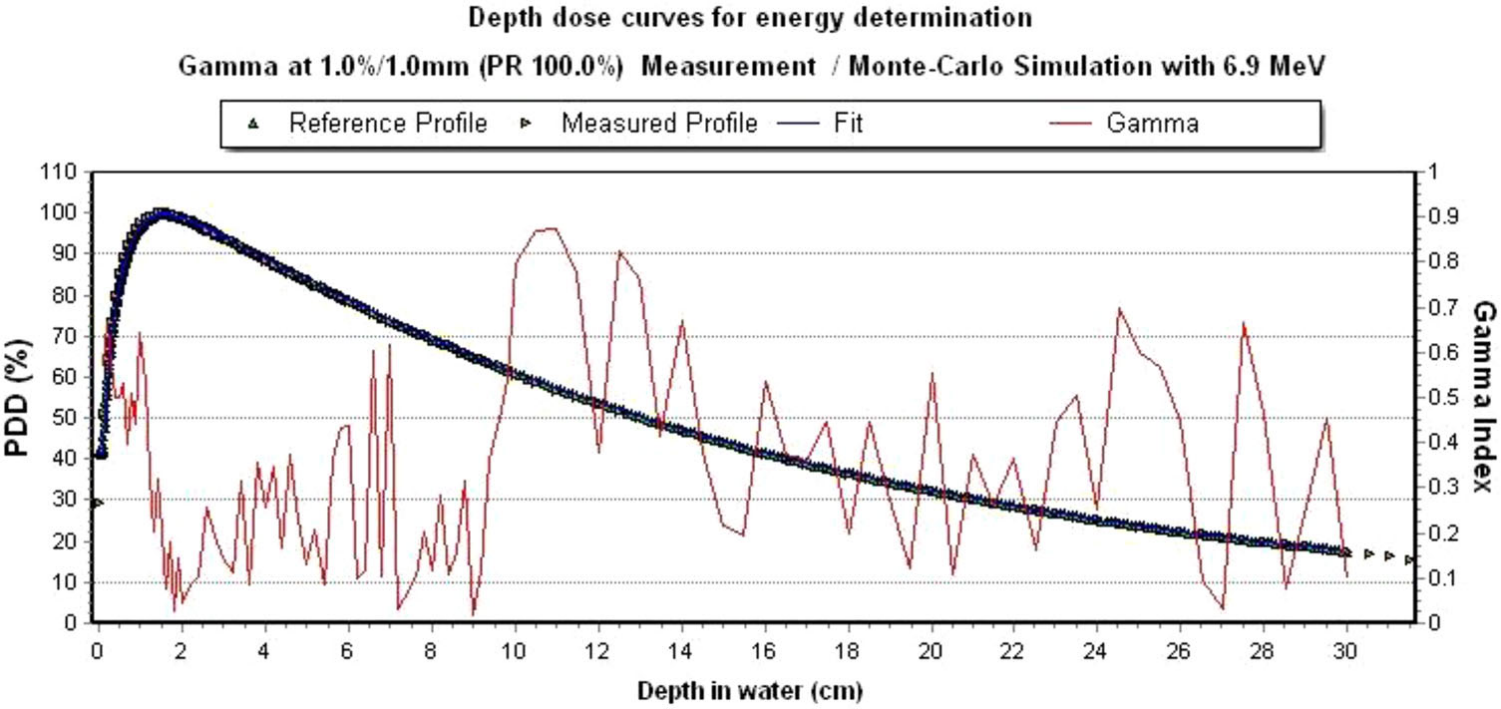
Energy determination of the CyberKnife VSI LINAC head: Comparison of the measured and simulated depth dose curves with a local gamma criterion of 1%/1 mm. The passing rate corresponds to 100%.

The average kinetic energy of 6.9 MeV was confirmed after the final source width was determined and the simulated dose profile agreed 100% with the measured dose profile of the 60 mm circular collimator at a global gamma criterion of 1%/1 mm. In Figure 5 is the final depth dose curve shown. The subsequent Monte Carlo simulations using EGSnrc were performed with an average monoenergetic energy of 6.9 MeV.

### 3.2 Final source width

In order to determine the final source width, several Monte Carlo simulations of dose profiles with a monoenergetic energy of 6.9 MeV were performed. The 60 mm circular collimator was used. The measurement depth is 15 mm below the water surface at a SAD of 800 mm. An adjustment of the FWHM was done in x‐ and y‐direction. The measured dose profile was compared with the simulated dose profiles from EGSnrc using the gamma analysis software ScanDoseMatch. The simulated source widths using EGSnrc in the x‐direction with the associated local gamma criteria are shown in Table 4.

**TABLE 4:** Source width and local gamma criterion with the respective passing rate in x‐direction.

*x_FWHM_* (cm)	3%/3 mm	2%/2 mm	1%/1 mm
0.20	100	92.3	42.3
0.21	100	96.2	53.8
0.22	100	100	76.0
0.23	100	100	80
0.24	100	100	92
0.25	100	100	100
0.26	100	100	96
0.27	100	100	84

**FIGURE 6:**
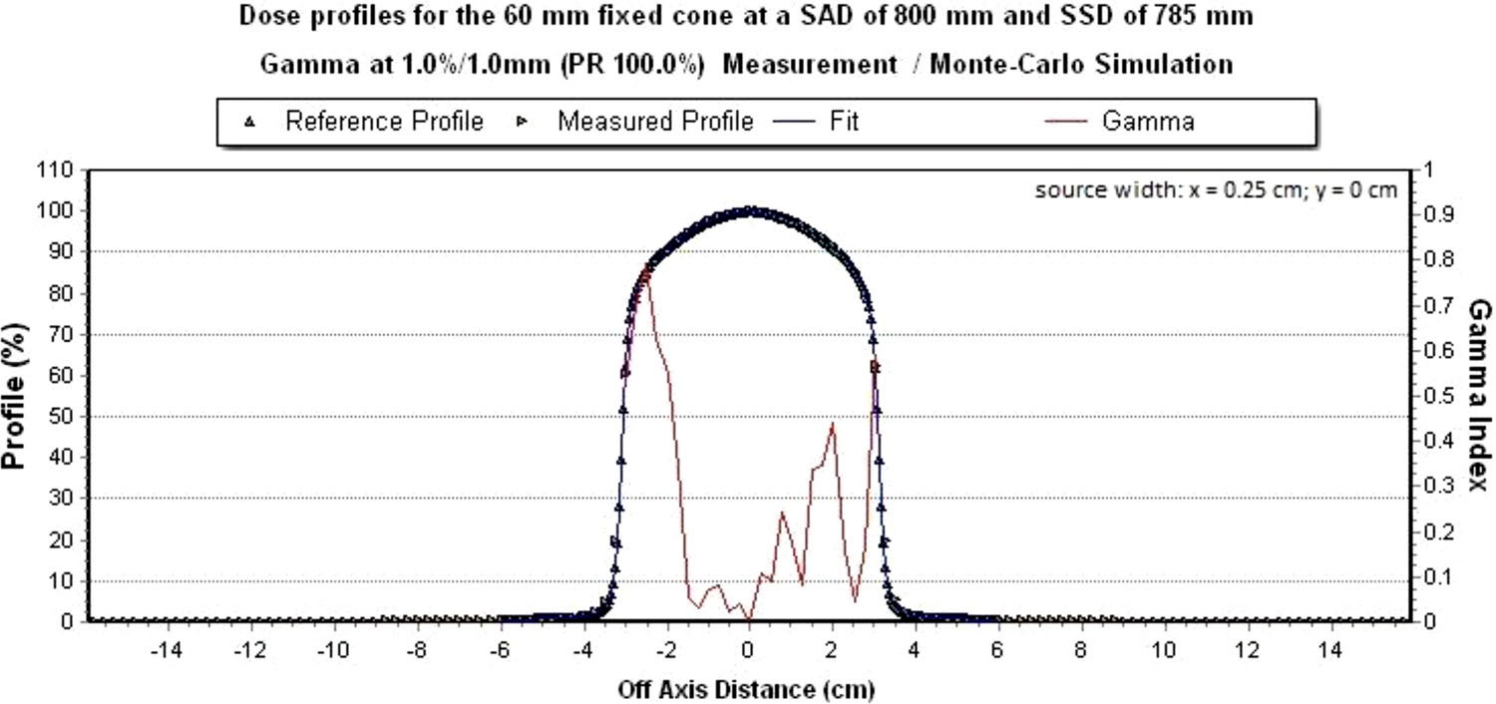
Simulated dose profile along the x‐axis with a final source width at which the FWHM in x‐direction is 0.25 cm. The local gamma criterion of 1%/1 mm is 100% met.

The FWHM with 0.25 cm in x‐direction alone fulfills the local gamma criterion 1%/1 mm and is thus the final source width in x‐direction. Figure 6 shows the dose profiles with the final source width in x‐direction.

Subsequently, the final source width in y‐direction was determined. The simulated source widths in y‐direction with the corresponding local gamma criteria are listed in Table 5.

**TABLE 5:** Source width and local gamma criterion with the respective passing rate in y‐direction.

*y_FWHM_* (cm)	3%/3 mm	2%/2 mm	1%/1 mm	0.5%/0.5 mm
0.21	100	100	96	–
0.22	100	100	96	–
0.23	100	100	100	84
0.24	100	100	100	76
0.25	100	100	100	68
0.26	100	100	80	–
0.27	100	100	84	–

**FIGURE 7:**
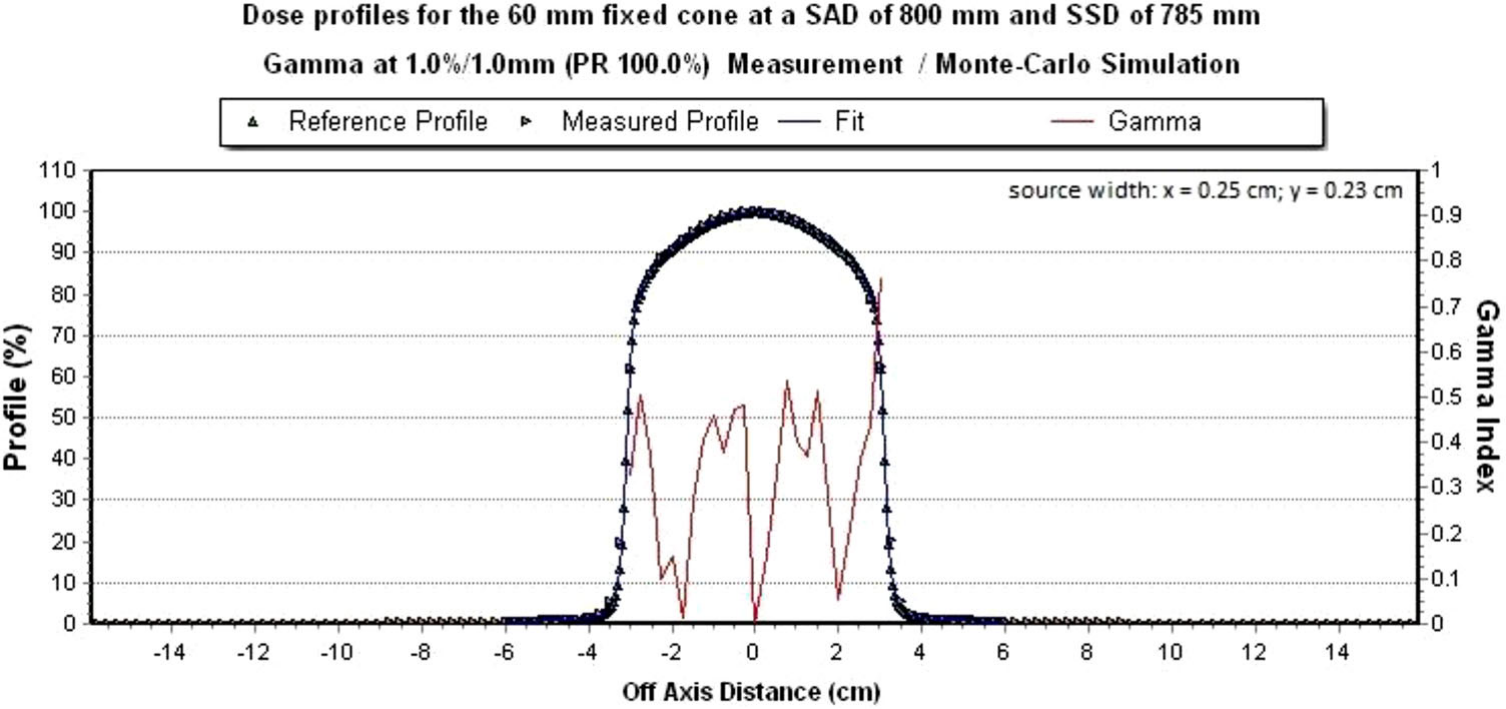
Simulated dose profile along the y‐axis with a final source width at which the FWHM in y direction is 0.23 cm. The local gamma criterion of 1%/1 mm is met 100%.

The FWHM with 0.23 cm in y‐direction has 100% at a local gamma criterion of 1%/1 mm. In addition, the FWHM with 0.23 cm at the local gamma criterion 0.5%/0.5 mm is the highest with 84 % compared to the FWHM with 0.24 and 0.25 cm. Figure 7 shows the measured dose profile with the simulated dose profile at a FWHM of 0.23 cm in the y‐direction.

With a final source width of 0.25 cm in x‐direction and 0.23 cm in y‐direction, further Monte Carlo simulations were performed in EGSnrc. The statistical uncertainty for all simulated dose profiles is ≤ 0.4% for dose values ≥ 20% and decreases towards the center of the central beam.

### 3.3 Dose profiles

The simulated dose profiles in EGSnrc for both collimation systems were examined with the measured dose profiles in ScanDoseMatch with respect to different gamma criteria. Tables 6 and 7 show that for all circular collimators the passing rate is 100% fulfilled for a global gamma criterion of 1%/1 mm.

**TABLE 6:** Results of gamma analysis of dose profiles for all circular collimators in x‐direction.

*x_Collimator_* (mm)	3%/3 mm	2%/2 mm	1%/1 mm
5	100	100	100
7.5	100	100	100
10	100	100	100
12.5	100	100	100
15	100	100	100
20	100	100	100
25	100	100	100
30	100	100	100
35	100	100	100
40	100	100	100
50	100	100	100
60	100	100	100

**TABLE 7:** Results of gamma analysis of dose profiles for all circular collimators in y‐direction.

*y_Collimator_* (mm)	3%/3 mm	2%/2 mm	1%/1 mm
5	100	100	100
7.5	100	100	100
10	100	100	100
12.5	100	100	100
15	100	100	100
20	100	100	100
25	100	100	100
30	100	100	100
35	100	100	100
40	100	100	100
50	100	100	100
60	100	100	100

**FIGURE 8:**
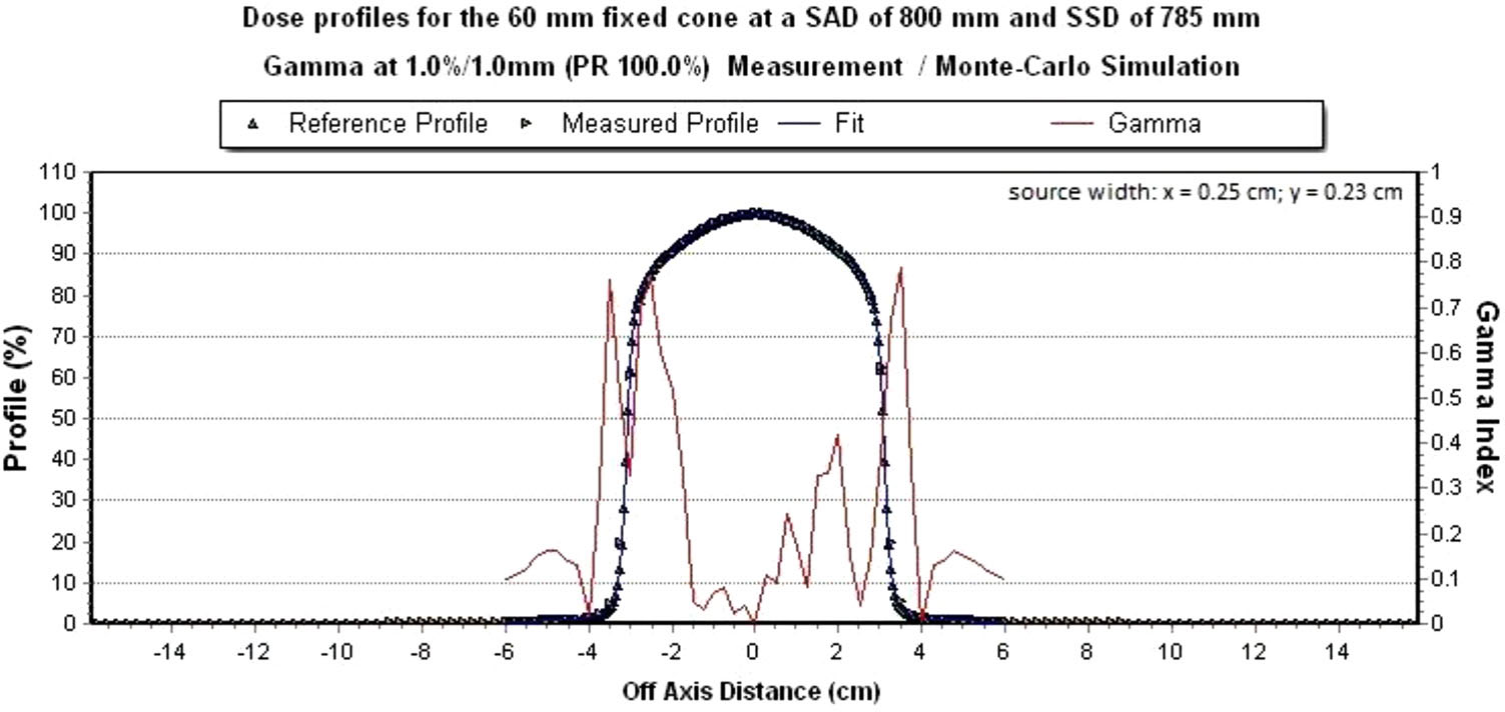
Simulated dose profile in x‐direction for the 60 mm circular collimator. The global gamma criterion 1%/1 mm is fulfilled to 100%.

**FIGURE 9:**
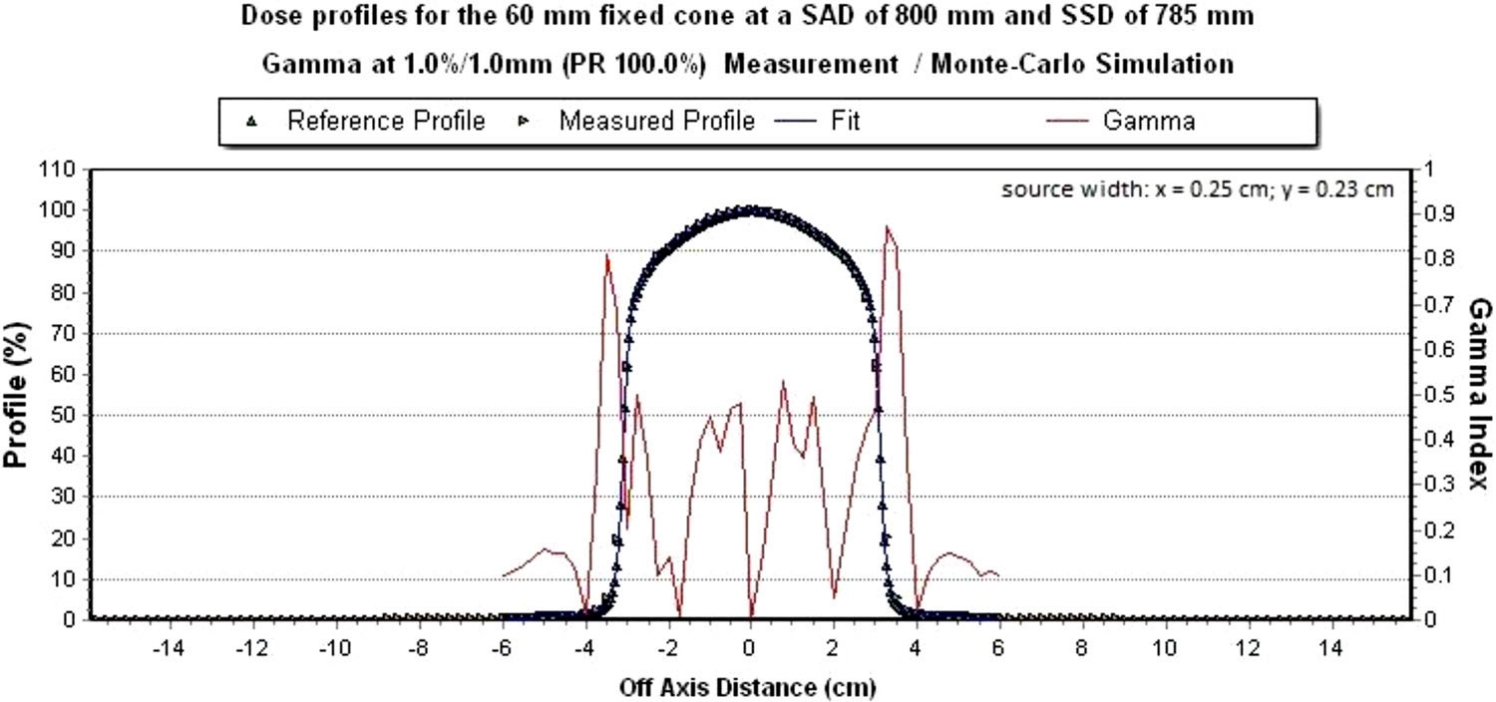
Simulated dose profile in y‐direction for the 60 mm circular collimator. The global gamma criterion 1%/1 mm is fulfilled 100%.

As an example, the measured dose profile with the simulated dose profile from the 60 mm circular collimator at a global gamma criterion of 1%/1 mm is shown below in Figures 8 and 9.

In the following Tables 8 and 9 are the results of the gamma analysis of the simulated dose profiles of the IRIS‐aperture.

**TABLE 8:** Results of gamma analysis of dose profiles for the IRIS‐aperture in x‐direction.

*x_Collimator_* (mm)	2%/2 mm	1%/1 mm
5	100	100
7.5	100	100
10	100	100
12.5	100	100
15	100	100
20	100	100
25	100	100
30	100	100
35	100	100
40	100	100
50	100	100
60	100	100

**TABLE 9:** Results of gamma analysis of dose transverse profiles for the IRIS‐aperture in y‐direction.

*y_Collimator_* (mm)	2%/2 mm	1%/1 mm
5	100	100
7.5	100	100
10	100	100
12.5	100	100
15	100	100
20	100	100
25	100	100
30	100	100
35	100	100
40	100	100
50	100	100
60	100	100

**FIGURE 10:**
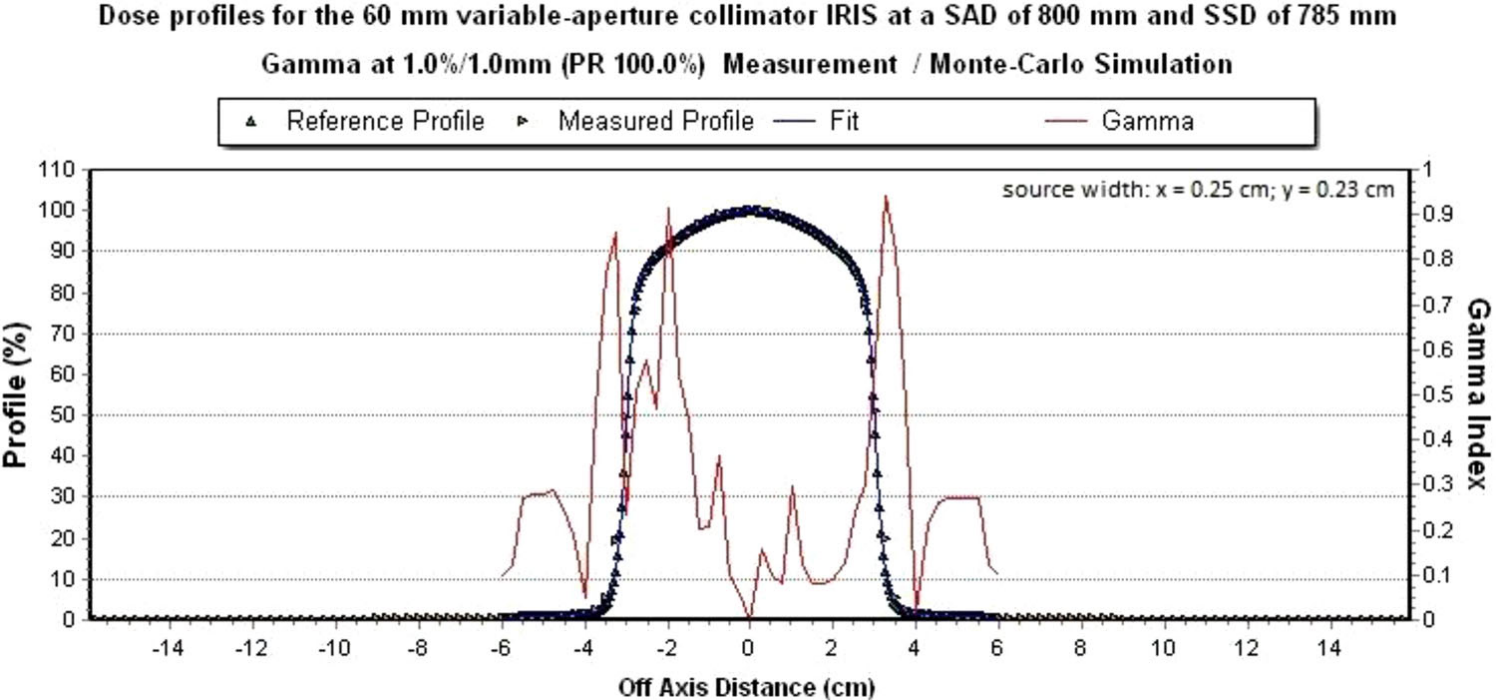
Simulated dose profile in x‐direction for the 60 mm IRIS‐aperture. The global gamma criterion of 1%/1 mm is met 100%.

**FIGURE 11:**
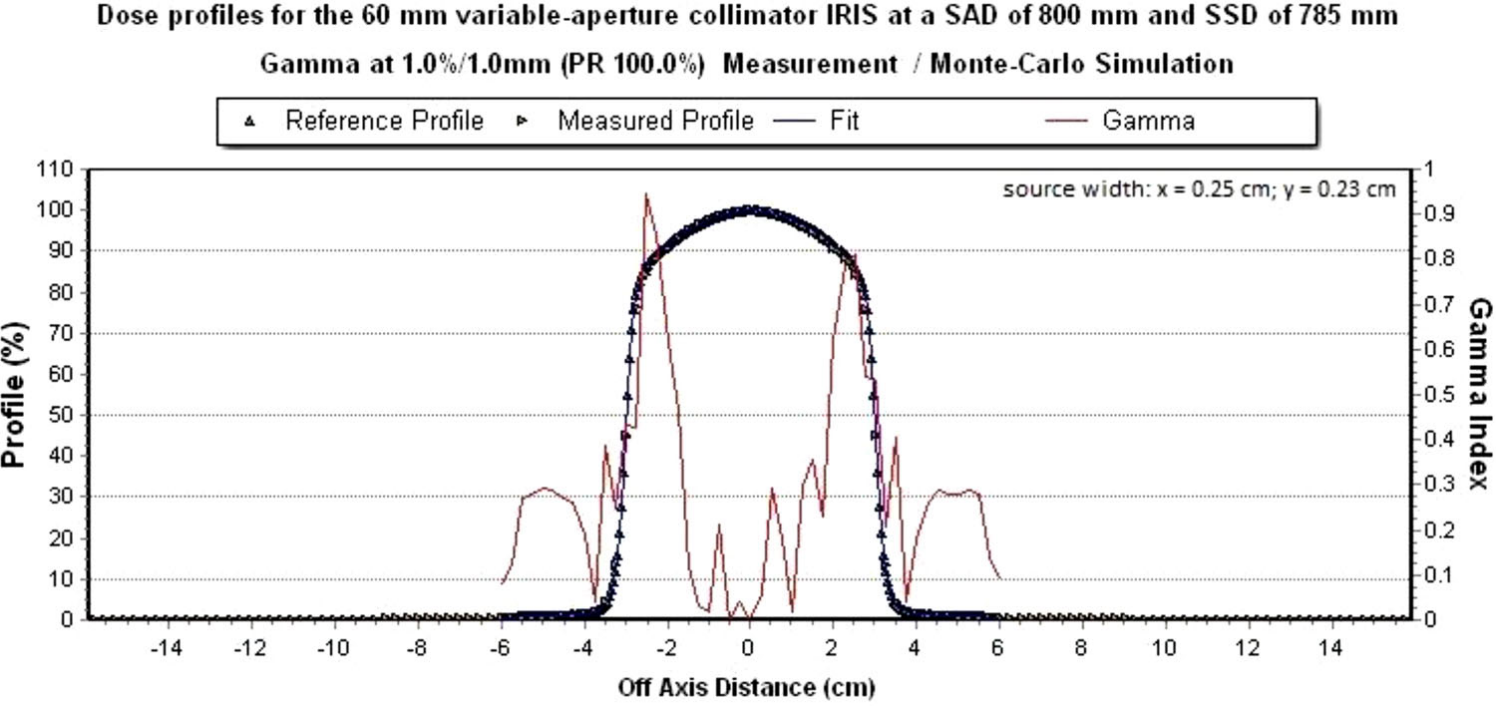
Simulated dose profile in y‐direction for the 60 mm IRIS‐aperture. The global gamma criterion of 1%/1 mm is met 100%.

All dose profiles of the IRIS‐aperture meet a global gamma criterion of 1%/1 mm in x‐ and y‐direction. Figures 10 and 11 show the measured dose profile with the simulated dose profile with a field size of 60 mm of the IRIS‐aperture at a global gamma criterion of 1%/1 mm.

The analysis of the half‐widths for the circular collimators and the IRIS‐aperture are summarized in Tables 10 and 11. Several Monte Carlo simulations using EGSnrc with field size adjustment had to be performed in order to achieve the desired deviation < 1% between the measured and the simulated dose profiles with respect to the half‐width. Only the 5 mm field size is above 1% for both collimation systems.

**TABLE 10:** Analysis of half‐width for all circular collimators.

FW (mm)	5	7.5	10	12.5	15	20	25	30	35	40	50	60
FWHM Measurement (mm)	5.23	7.78	9.98	12.48	15.10	20.23	25.29	30.56	35.60	40.80	50.90	61.15
FWHM MC Simulation (mm)	5.32	7.8	9.97	12.48	15.12	20.41	25.30	30.60	35.87	41.03	51.29	61.38
Difference (%)	−2.25	−0.22	0.12	−0.02	−0.15	−0.91	−0.04	−0.13	−0.78	−0.55	−0.77	−0.38
Difference (mm)	−0.12	−0.02	0.01	0.00	−0.02	−0.18	−0.01	−0.04	−0.28	−0.22	−0.39	−0.23

**TABLE 11:** Analysis of half‐width analysis for the IRIS‐aperture.

FW (mm)	5	7.5	10	12.5	15	20	25	30	35	40	50	60
FWHM Measurement (mm)	5.15	7.80	10.20	12.61	15.12	20.12	25.03	29.98	34.69	39.66	49.74	59.53
FWHM MC Simulation (mm)	5.50	7.76	10.23	12.61	15.02	19.98	24.85	29.79	34.44	39.53	49.68	59.56
Difference (%)	−6.93	−0.45	−0.25	0.00	0.63	0.68	0.73	0.61	0.72	0.31	0.11	−0.05
Difference (mm)	−0.36	−0.04	−0.03	0.00	0.10	0.14	0.18	0.18	0.25	0.12	0.05	−0.03

The statistical uncertainty for all simulated dose profiles of both collimation systems is ≤ 0.4% for dose values ≥ 20% of the respective collimator size and decreases towards the center of the central beam. The smaller the field size, the lower the statistical uncertainty. For a field size of 5 mm, this is ≤ 0.1%.

### 3.4 Output factors

The simulated output factors, which were performed with a monoenergetic energy of 6.9 MeV and a final source width (FWHM 0.25 cm in x‐direction and 0.23 cm in y‐direction) for each collimator, are listed in Table 12 for circular collimators and in Table 13 for the IRIS‐aperture. For collimator sizes ≥ 12.5 mm and larger for the circular collimators, and for collimator sizes ≥ 10 mm and larger for the IRIS‐aperture, the percent deviation is less than 1%. For collimator sizes < 12.5 mm for the circular collimators and < 10 mm for the IRIS‐aperture, the percent deviation is greater than 1%. The percentage deviation is largest for the 5 mm collimator size. The statistical uncertainty for both collimation systems is 0.5% for a FW of 60 mm– 50 mm, 0.4% for a FW of 40 mm, 0.3% for a FW of 35 to 30 mm, 0.2% for a FW of 25 to 15 mm, and 0. % for a FW ≤ 12.5 mm.

**TABLE 12:** Comparison of measured with simulated output factors for all circular collimators.

FW (mm)	5	7.5	10	12.5	15	20	25	30	35	40	50	60
FWHM Measurement (mm)	0.6841	0.8333	0.8823	0.9199	0.9426	0.9660	0.9759	0.9823	0.9874	0.9907	0.9955	1.0000
FWHM MC Simulation (mm)	0.6102	0.8198	0.8718	0.9176	0.9428	0.9668	0.9797	0.9835	0.9860	0.9911	0.9960	1.0000
Difference (%)	10.80	1.62	1.19	0.25	−0.02	−0.09	−0.39	−0.12	0.14	−0.05	−0.05	0.00

**TABLE 13:** Comparison of measured with simulated output factors for the IRIS‐aperture.

FW (mm)	5	7.5	10	12.5	15	20	25	30	35	40	50	60
FWHM Measurement (mm)	0.5503	0.7906	0.8716	0.9118	0.9358	0.9612	0.9724	0.9786	0.9837	0.9860	0.9906	0.9951
FWHM MC Simulation (mm)	0.4173	0.7772	0.8716	0.9141	0.9403	0.9672	0.9784	0.9830	0.9903	0.9877	0.9919	0.9951
Difference (%)	24.17	1.69	0.00	−0.25	−0.49	−0.62	−0.62	−0.45	−0.67	−0.18	−0.13	0.00

## 4 DISCUSSION

For both collimation systems, agreement between the measured data and simulation data was obtained using Monte Carlo simulation with EGSnrc system. The self‐defined gamma criterion of 1%/1 mm for the depth dose curves and for the dose profiles could be fulfilled. Thus, a harder criterion was chosen than 2%/2 mm, as recommended and described in the literature.^20^, ^25^, ^26^ Due to the small effect on the half width, a change of the energy and the scattering angle was omitted.^20^, ^27^ Mackeprang et al., who modeled the CyberKnife M6 with multileaf collimator using EGSnrc, could show that a gamma criterion of 2.3%/1 mm between measured data and simulation data can be kept (except for the small field sizes). Concerning the kinetic energy of the electron beam, Araki, Francescon et al. and Moignier et al. came to a similar result with 6.7 and 7 MeV, as in the present work.^9^, ^27^, ^28^ This result is also present for the final source width in the x‐ and y‐directions (FWHM: 0.21–0.24 cm).^27^, ^28^ However, the models of Araki, Francescon et al. and Moignier et al. do not include IRIS‐aperture. In Figure 5, an increase in gamma value can be seen from a water depth of 10 cm, although it should remain the same. This result is apparently because the voxels were not chosen to be equidistant. The analysis of the field size in both collimation systems between measured data and simulation data shows a deviation < 1%. However, if one wants to have a smaller deviation, the symmetrical fitting of the circular collimators can lead to a problem, since the dose profile may shift in the x‐ or y‐direction. The same problem applies to the IRIS‐aperture. The component modules upper and lower bank (BankUp and BankLow) are point symmetric. For the output factors, a deviation from simulation to measurement data of < 1% was found for a field size of 12.5 mm for the circular collimators and for a field size of 10 mm for the IRIS‐aperture. For both collimation systems, the largest deviation of the output factor is present at a field size of 5 mm. Since the 5 mm field size is not used at the German CyberKnife‐Center in Soest, it was not investigated further. According to AAPM Report 157, a maximum deviation of 2% between measured and simulated data is allowed.^25^ This would be given for all simulated field sizes ≥ 7.5 mm for both collimation systems.

## 5 CONCLUSION

The average kinetic energy for the CyberKnife VSI LINAC head is 6.9 MeV. The final source width is 0.25 cm in x‐direction and 0.23 cm in y‐direction. For the dose profiles, a global gamma criterion of 1%/1 mm can be met for both collimation systems with respect to simulated data to measured data. The simulated field sizes have a deviation < 1% for both collimation systems at a field size ≥ 7.5 mm. For the output factors, the deviation from simulated to measured data is < 1% from a field size of 12.5 mm for the circular collimators and from a field size of 10 mm for the IRIS‐aperture. It could be shown that by geometric adjustment of the field size in EGSnrc the simulation data can correspond to the measured data. With the created model, it is now possible to perform an electronic patient‐specific QA or to check other research fundamentals, such as scatter radiation processes.

## AUTHOR CONTRIBUTIONS

Martin Thiele: Conceptualization (lead), Formal Analysis (lead), Investigation (lead), Methodology (lead), Software (lead), Validation (lead), Visualization (lead), Writing and Original Draft Preparation (lead), Writing and Review.

## CONFLICT OF INTEREST STATEMENT

The authors declare no conflicts of interest.
